# (In)comparability of Carotid Artery Stent Characteristics: A Systematic Review on Assessment and Comparison with Manufacturer Data

**DOI:** 10.1007/s00270-020-02499-1

**Published:** 2020-05-14

**Authors:** Evelien E. de Vries, Mert Kök, Astrid M. Hoving, Cornelis H. Slump, Raechel J. Toorop, Gert J. de Borst

**Affiliations:** 1 Department of Vascular Surgery, University Medical Center Utrecht, Room G04.129, PO Box 85500, 3508 GA Utrecht, The Netherlands; 2 Department of Robotics and Mechatronics (EEMCS), University of Twente, PO Box 217, Enschede, The Netherlands

**Keywords:** Carotid artery stent, Carotid stenosis, In vitro testing, Mechanical behavior, Systematic review

## Abstract

**Purpose:**

Carotid stent (CS) characteristics, such as radial force, scaffolding and flexibility, are continuously modified by stent manufacturers aiming to improve stent performance. Since manufacturers’ definitions and assessment methods are not disclosed, it is unknown how characteristics of different CSs relate to each other or to published literature. We examined in vitro methodological techniques used to measure CS characteristics and assessed comparability between published papers and outcomes as provided by the manufacturers.

**Methods:**

A systematic review was conducted in MEDLINE, Embase, Cochrane, and Scopus databases. Studies reporting on in vitro investigations of predefined characteristics of CS used in current everyday clinical practice were included. The predefined characteristics were radial force, scaffolding, flexibility, foreshortening, side-branch preservation and visibility. Eight manufacturers of 10 currently used CS were contacted and data on the predefined device characteristics was requested.

**Results:**

12 published articles were included and six stent manufacturers provided data on six stents (two refused to share data). Used methodologies to measure stent characteristics in published literature and manufacturer data varied greatly for all included characteristics except foreshortening. The number of different units of measurement to express outcomes ranged from two for foreshortening to six for radial force.

**Conclusion:**

A variety of methodologies and outcome measures is used to quantify CS characteristics, which hampers comparisons between published studies and manufacturer data. Future studies are encouraged to synchronize methodologies and outcome measures. Manufacturers are encouraged up to increase transparency of applied testing methodologies and outcomes.

**Electronic supplementary material:**

The online version of this article (10.1007/s00270-020-02499-1) contains supplementary material, which is available to authorized users.

## Introduction

Carotid artery stenting is a minimally invasive alternative to carotid endarterectomy for treatment of significant carotid artery stenosis. However, as the 30-day (minor) stroke rate as well as the rate of subclinical ischemic events is currently to the detriment of carotid stenting, this treatment is reserved for the patients at deemed high surgical risk [1]. Since the majority of 30-day strokes occur on the day of the procedure [2], it is likely that short-term outcomes of carotid stenting can be improved by improving the procedural aspects, including the stent, itself.

Stent manufacturers continuously apply changes to their devices in order to achieve acclaimed improvement of carotid artery stent (CS) characteristics and stent performance, and hereby potentially aid in prevention of procedural strokes. However, although device characteristics such as radial force and flexibility can be obtained from stent manufacturers, these characteristics are not commonly provided in the product description. In addition, most often the applied methods to measure these characteristics are not disclosed. Several experimental studies have been published which aimed to compare characteristics between stents [3–5]. However, it is unknown how these industry-independent papers relate to each other in terms of tested devices, device characteristics, and applied measurement methods. At the end, it is currently unknown to what degree clinicians can draw conclusions from stent characteristics provided by the manufacturers, which might hamper adequate patient-device matching.

Therefore, the aim of this study was to examine and compare in vitro methodological techniques used to measure CS characteristics and assess comparability between published papers and outcomes as provided by the manufacturers.

## Methods

### Systematic Review

This systematic review was conducted in accordance with the Preferred Reporting Items for Systematic Reviews and Meta-Analyses (PRISMA) statement.

#### Search Strategy and Study Selection

In order to identify in vitro measurement techniques of carotid artery stent characteristics, a systematic literature search was conducted in MEDLINE, EMBASE, Cochrane, and Scopus databases in May 2018. The Medical Subject Headings (MeSH) terms for ‘carotid artery,’ ‘stent,’ and ‘in vitro techniques’ were combined with synonyms for various stent characteristics. The predefined stent characteristics included radial force, scaffolding, flexibility, foreshortening, side-branch preservation, and visibility. The full search strategy can be found in the Supplementary material (file 1).

Included were studies that tested: (1) any predefined stent characteristics, (2) carotid artery stents used in current everyday clinical practice, (3) in vitro experimental set-ups (hence, computational modeling set-ups were excluded). A recently published worldwide meta-analysis was used to select the stents that had been used regularly in the previous 5 years [6]. The following stents were included: Acculink and Xact (Abbott Vascular, Santa Clara, CA, USA), Precise (Cordis, Miami Lakes, FL, USA), Wallstent (Boston Scientific, Marlborough, MA, USA), Protégé and Cristallo Ideale (Medtronic, Fridley, MN, USA). Four relatively recently clinically introduced stents were added to guarantee topicality: Roadsaver/CASPER-RX (Terumo corp., Tokyo, Japan/Microvention), CGuard (InspireMD, Boston, MA, USA), Gore carotid stent (Gore, Flagstaff, AZ, USA), and Cardiatis flow modulator stent (Cardiatis, Isnes, Belgium) [7]. Exclusion criteria were: reviews, animal studies, computer simulation studies, and papers not written in English.

All titles and abstracts were independently screened by two investigators (MK, EEV), and full-text eligibility was assessed. Judgment differences were resolved by discussion. Reference lists of included papers were scrutinized for missing articles.

NB. The terms open and closed cell stent are commonly used in the literature and refer to the scaffolding properties of the stent (open cell, larger free cell area with fewer interconnections; closed cell, smaller free cell area with more interconnections). In this paper, we adhere to the definition as provided by the manufacturers and used in the included papers.

#### Data Extraction

Prespecified descriptive variables and quantitative outcomes were recorded in a spreadsheet, including: study characteristics (year of publication, study design), type of tested stents, studied stent characteristics, experimental methodology, and study outcomes.

### Manufacturer Data

Websites of the manufacturers (eight manufacturers for 10 selected stents) were thoroughly checked for the desired information (Supplementary material, file 2): their definition of the characteristic, the applied measurement method, and experimental outcomes. None of the websites revealed any of the desired information. The manufacturers were subsequently contacted and asked to provide the information on each of the stent characteristics. A predefined contact form was used (Supplementary material, file 3).

## Results

### Search Results Systematic Review

**Fig. 1:**
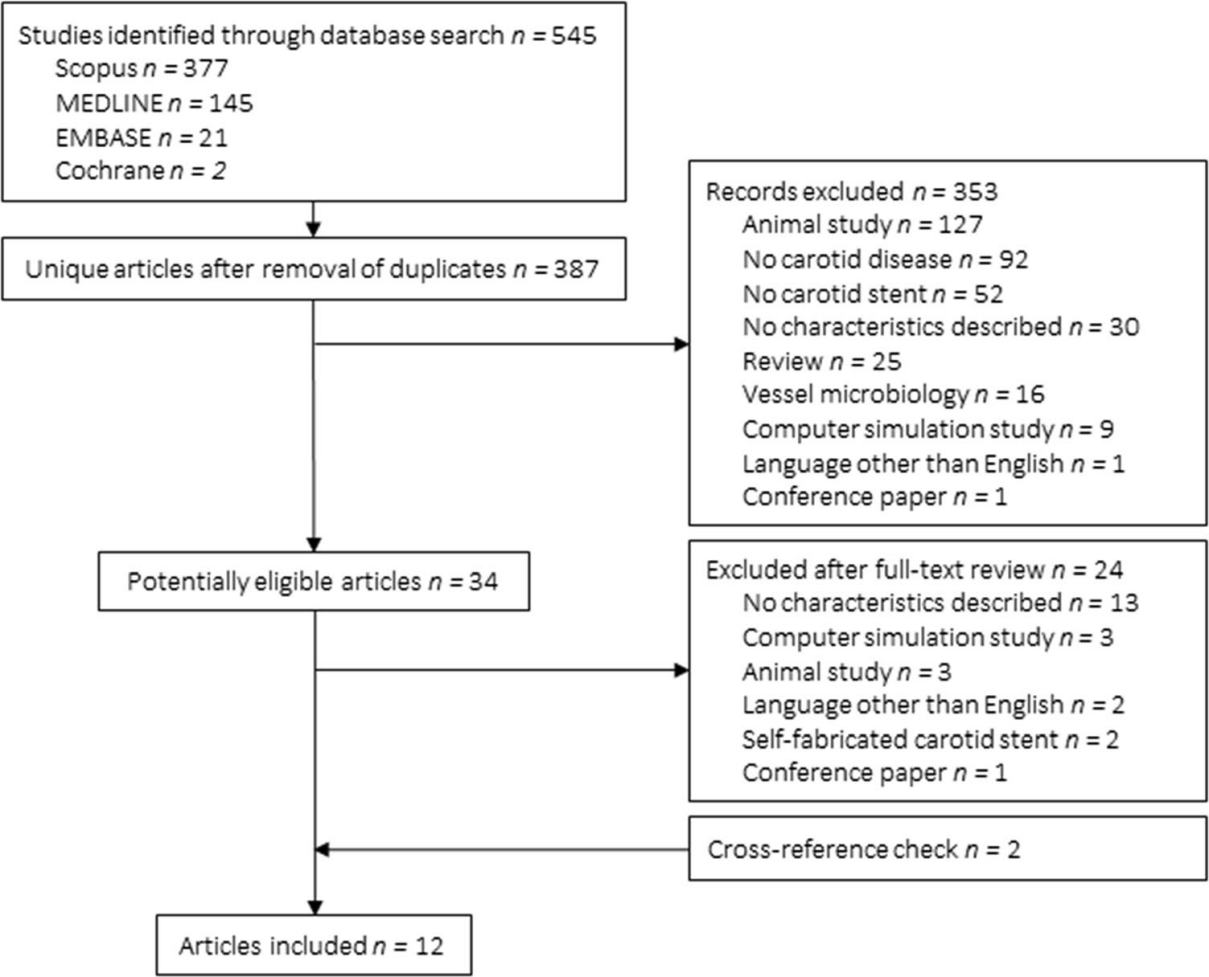
Flowchart of search strategy

The search yielded 545 articles, of which 34 were retrieved for more detailed evaluation (Fig. 1). Some 12 studies met the inclusion criteria and were included. Table 1 presents an overview of the characteristics that have been tested and the measurement methods.

**Table 1:** Literature overview: tested device characteristics, their definitions, the number of studies that measured the characteristic, and study methodology

Stent characteristics^a^	Definition	No. of studies	Methodology
Flexibility	The bending or torsion stiffness of the stent.	6	*Bending* 1. 4-point bending test: max. deflection and bending stiffness calculated based on measured force applied by testing machine and crosshead displacement [9] 2. 3-point bending test: measurement of bend load required to flex the stent 25° [8] 3. Stent fixed on one end. Measurement of force needed to bend stent 20°–30° [3], or force needed to create max. bending deformation of 5 mm [4, 10, 11], the latter complimented with stent mounted on delivery system vs expanded in 7 mm vascular model [10] *Torsion* 1. Stent fixed on one end. Measurement of force or rotation load required to rotate stent 10°–15° [3], or 30° [8]
Conformability	The ability of the stent to conform to the geometrical shape of the artery.	4	Stents implanted in pulsatile [12] or rigid [4, 10, 11] carotid models 1. Assessment of changes in shape/course of the artery on X-ray, and dehiscences between stent filaments and arterial wall on DSA [12] 2. Conformability visually assessed on fluoroscopic images [4, 10, 11]
Radial force	The force needed for stent compression or collapse.	5	1. Stent placed into V-shaped support system using a 3-point test. Stent resistance to local compression was measured (relationship between applied force and crosshead displacement of 0.1–0.5 mm) [9] 2. Force necessary for stent compression was measured (until 1/3rd of fully expanded diameter) after compression between two parallel plates, and circumferential compression by using a radial closing device [4] 3. Stents were deployed into a thin flexible tube, placed in a pressure chamber and a hydrolic radial load was applied. Collapse pressure was measured (until cross section of stent was 50% of initial state) [4, 10, 11]
Outward pressure	The pressure exerted by the stent onto the vessel at a certain level of expansion.	5	1. A thin film is looped around the stent. On loading the loop decreases in diameter and stent is circumferentially compressed. Chronic outward force is measured at max. − 1 mm of expanded state [13] or at 3 mm expansion [5] 2. Measurement of resulting force exerted by the stent on prismatic clamping supports during expansion to 5–7 mm [4, 10, 11]
Visibility	The degree to which the stent itself or in-stent area is assessable on post-procedural imaging.	3	1. Multiple stents were implanted in vascular phantoms, images were acquired on (CE)MRA system. Visibility graded with scoring system based on (1) signal intensity, lumen narrowing, and lumen homogeneity [14] (2) signal loss within stent and artificial lumen narrowing, in-stent patency and in-stent stenosis [15] 2. Stent placed in plexiglas phantom, grading of absorption value on X-ray [13]
Foreshortening	The difference in stent length before and after stent expansion.	2	1. Stent length measured while mounted on delivery system and after stent release in completely expanded state [4], complemented by stent release in vessel models of 5–7 mm inner diameter [10]
Scaffolding	The amount of coverage the stent provides to the vessel wall and lesion site.	1	1. (a) Metal-to-artery ratio was calculated by determining max. number of max. radius fitted-in circles, (b) stents were inserted into a silicone tube, 8 different plastic spheres of 1.5–6.0 mm were positioned onto the stent and pushed through the pores with measured force [3]
Side-branch preservation	The influence of the deployed stent on the blood flow to the side-branches.	1	1. Stents were deployed in silicone carotid artery bifurcation models. Flow measurements were performed with laser Doppler anemometry (LDA), using pulsatile flow conditions [16]

^a^Synonyms used in papers: *Radial force* radial stiffness, collapse pressure, hoop strength; *Visibility* radiopacity; *Flexibility* bending stiffness, bending force, torsion; *Outward pressure* radial force, chronic outward force, radial resistive force; *Conformability* conformity, wall adjustment, wall adaptation

### Manufacturer Data

Data were provided by six of eight contacted manufacturers for six stents. Two manufacturers did not provide data despite personal contact (Abbott for Acculink and Xact, and Cardiatis for Cardiatis flow modulator). One stent was withdrawn from the manufacturers’ portfolio (Cristallo Ideale, Medtronic). Detailed information on experimental methodology and outcomes as provided by the manufacturers can be found in Supplementary material (file 4, Tables 1–6).

### Stent Characteristics

**Fig. 2:**
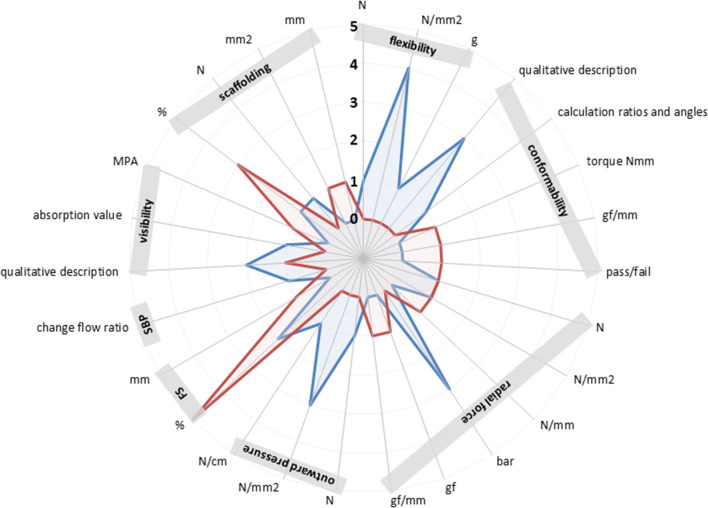
Radar graph of the included stent characteristics, represented separately for published papers (blue) and manufacturer data (red). Each axis of the circular radar graph represents one unit of measurement, which are grouped per characteristic. The *y*-axis represents the number of studies that use the measurement unit. *FS* foreshortening, *SBP* side-branch preservation

Below, for each characteristic, first an overview of the literature is given, followed by the data as provided by the manufacturers. Figure 2 provides an overview of the included stent characteristics and accompanying units of measurement in order to illustrate the differences in usage of units of measurement (and thus methodology). A distinction was made between published papers and manufacturer data.

#### Flexibility and Conformability

##### Literature on Flexibility

The flexibility of the stent was the most commonly assessed device characteristic as it was studied in six papers (Table 1) [3, 4, 8–11]. Outcomes were recorded in Newton, or Newton per mm^2^ or grams. Two studies, both using a similar set-up, found lower flexibility in closed cell stents compared to open cell stents, with the hybrid Cristallo Ideale stent lying in between [3, 4]. In contrast, the studies using the 3- or 4-point bending test found lower flexibility for the open cell stents Acculink and Protégé [8, 9]. Additionally, one of the latter studies demonstrated relatively high flexibility for the closed cell Wallstent [8]. Measurements of the double mesh stents CASPER–RX and CGUARD have been reported in one paper. The Roadsaver had a relatively low flexibility resembling Wallstent (if compared to a previous study with similar set-up) [10]. The flexibility of CGUARD was very low when mounted on the delivery system, but significantly higher in expanded state [11].

##### Literature on Conformability

Conformability of the stents was studied in four papers (Table 1) [4, 10–12]. Outcomes were either qualitatively appraised based on visual assessment [4, 10–12], or quantified by means of the dehiscence ratio (area between the struts and the vessel wall) and differences in pre- and post-stent situation (e.g., offset angulation of the internal carotid artery) [12]. Wallstent was the only stent being included in more than one paper. Both papers showed limited adaptation for Wallstent [4, 12], with concomitant vessel straightening [12]. Other conventional stents were investigated in one and the same paper, showing ‘good’ adaptation for the open cell stents Precise and Protégé and the hybrid Cristallo Ideale stent [4]. For the two double mesh stents, the diameter confirmation of CGUARD was superior to Roadsaver [10, 11].

##### Manufacturer Data

Data on conformability or flexibility were provided by four manufacturers (for Wallstent, Protégé, CGUARD and Roadsaver, Supplementary material, file 4—Table 1). However, for CGUARD a reference to a conference presentation was given. Outcomes were provided in torque Newton mm, gram-force per mm, or scored as pass/fail. Protégé was tested using the 3-point bending test similar to two published studies [8, 9]. However, it was unknown to what degree the stent was flexed, and the outcome was recorded in gram-force per mm instead of Newton per mm^2^ or grams. No other comparisons with published literature were possible given the heterogeneity in methodology and outcome recordings.

#### Radial Force and Outward Pressure

##### Literature on Radial Force

Radial force of the devices (hence, the pressure the stent can withstand) was measured in five studies (three articles from the same research group) [3, 4, 9–11]. Radial force of Xact and Cristallo Ideale was shown to be the lowest in two studies with a different set-up [3, 4]. Wallstent on the other hand had average to high radial force [3, 4], which was also true for Protégé [3, 4, 9]. Acculink had average radial force in two papers [3, 9], while results on Precise stent were inconsistent [3, 4, 9]. Roadsaver and CGUARD stents had high and average radial force, respectively [10, 11].

##### Literature on Outward Pressure

The pressure exerted by the stent onto the vessel wall was studied in five papers (Table 1) [4, 5, 10, 11, 13]. The open cell stents Precise and Protégé exerted the highest outward pressure, while Acculink had moderate outward pressure, and the closed cell stents Wallstent and Xact had the lowest outward pressure [4, 5]. Roadsaver exerted relatively low outward pressure [10], while CGUARD exerted a high outward pressure which was comparable to Precise and Protégé [11].

##### Manufacturer Data

Manufacturers were asked to provide data on radial force of the stents (Supplementary material, file 4—Table 2). One manufacturer did not provide the data (for Gore carotid stent) and one manufacturer referred to a conference presentation (for CGUARD). Three manufacturers provided the outward pressure exerted by the stent (for Protégé, CGUARD and Roadsaver), one provided the radial force (collapse pressure, for Precise), and in one case it was unsure whether radial force or outward pressure was provided (for Wallstent). Outcomes were diversely recorded in gram-force, gram-force per mm, Newton per mm, or Newton per mm^2^. This hampered direct comparisons between manufacturers and comparisons with published literature. Only outward pressure of Protégé was expressed in similar units as one published study, and seemed comparable (0.075 N/mm^2^ vs 0.064 N/mm^2^) [4].

#### Visibility

##### Literature on Visibility

Three different studies investigated stent visibility (Table 1). Acculink had ‘excellent’ visibility of stent patency and accuracy of grading in-stent stenosis in one study [15], while another showed ‘intermediate’ visibility in comparison to other stents (but none of them involving currently used CSs) [13]. Wallstent had an ‘intermediate’ absorption value in one paper [13].

##### Manufacturer Data

Five manufacturers provided data on stent visibility (Supplementary material, file 4—Table 3). However, for Precise and CGUARD no measurement methods were precluded. For Wallstent and Roadsaver, radiopacity was calculated and expressed in MPA or graded on a 3-point performance scale. For Protégé and Precise, only a description of visible markers on stent or delivery system was given.

#### Foreshortening

##### Literature on Foreshortening

The change in stent length before and after deployment was studied in two papers from the same research group [4, 10]. Strongest foreshortening was seen in Roadsaver (28% length change) and Wallstent (22%) [4, 10]. Precise, Xact and Protégé stents showed minimal foreshortening (6%, 3% and 2%, respectively), while Cristallo Ideale showed virtually no foreshortening (0.5%) [4].

##### Manufacturer Data

All manufacturers provided data on foreshortening, which was uniformly defined as the change in length before and after deployment (Supplementary material, file 4—Table 4). In all but one, the outcome was given as a percentage. Manufacturer outcomes were comparable to published literature for Precise and Protégé [4], and for Roadsaver [10]. Foreshortening of Wallstent was more pronounced when measured by the manufacturer as compared to the literature (49% vs 22%, respectively) [4].

#### Scaffolding

##### Literature on Scaffolding

One paper investigated scaffolding capacities of six relevant CSs [3]. The open cell stents Acculink, Precise, and Protégé had lowest resistance to particle penetration, and Acculink also allowed the largest spheres (6.0 mm) to pass. The closed cell Xact and hybrid Cristallo Ideale only allowed the smallest spheres (1.5–2.0 mm) to pass. Wallstent, although a closed cell stent with highest metal-to-artery ratio, allowed particle penetration of medium sized spheres due to the mesh design with wires capable of moving.

##### Manufacturer Data

All manufacturers provided data on the scaffolding properties of the stents (Supplementary material, file 4—Table 5). For Wallstent, Protégé and Roadsaver, the metal-to-artery ratio was provided. The metal-to-artery ratio of Protégé was higher according to the manufacturer when compared to the ratio found in the literature (29% vs 19%, respectively) [3]. In contrast, for Wallstent the manufacturers’ metal-to-artery ratio was lower when compared to outcomes of the same published paper (15% vs 21%, respectively). For Precise, CGUARD, and Gore only a description of the pore size was provided, which was significantly smaller for the double mesh stents (8.2 mm^2^, 150–180 μm, and 500 μm, respectively). For Wallstent, the maximum cell diameter was provided as well (1.7 mm).

#### Side-Branch Preservation

##### Literature on Side-Branch Preservation

Side-branch preservation was studied in one paper [16], which measured hemodynamic changes in the blood flow in a carotid bifurcation phantom, before and after stent placement. Wallstent was the only CS included. The flow rate ratio in the external versus internal carotid artery remained nearly unchanged [16].

##### Manufacturer Data

For Wallstent and Protégé, manufacturers provided data on the metal-to-artery ratio (Supplementary material, file 4—Table 6), while for Precise a general product description was provided, and for CGUARD and Gore carotid stent a reference to studies that evaluated in vivo external carotid artery patency was given. Manufacturers of Roadsaver evaluated side-branch flow in an animal laboratory study and found ‘excellent’ flow at 1 year follow-up.

## Discussion

This study evaluated in vitro methodological techniques used to measure carotid artery stent characteristics, and assessed comparability between published papers and outcomes as provided by manufacturers. Of the six characteristics compared, only foreshortening was defined and measured similarly in published papers and in manufacturers’ experiments. A variety of methodologies and outcome measures was described for the other device characteristics, which hampered comparisons between published literature and manufacturer data.

For carotid artery stenting to succeed, the carotid stent needs to be capable of sufficient plaque coverage, but also has to have adequate levels of flexibility (during maneuvering of the stent delivery system), conformability (to the vessel curvature), and radial force (to withstand pressure from outside) [17]. In general, due to higher flexibility so-called open cell stents are reserved for tortuous anatomies, while closed cell stents are used in patients with vulnerable plaques due to better scaffolding capacities (at the expense of lower flexibility) [7]. Our paper illustrates several problems that are currently at hand:

First of all, in order to adequately compare the stents and their characteristics, either the methodological set-up between papers should be comparable, or multiple devices need to be tested in one paper. Unfortunately, comparisons between papers which measure stents were largely impossible due to the heterogeneous methodology, which is also recognized by one paper mentioning that direct quantitative comparisons were hampered by the differing test methods [11]. There were several papers which included multiple stent designs in their experiments. They partially confirmed the aforementioned concept of design-dependent characteristics [3, 4]. Still, this distinction of open and closed cell stents seems to be too simplistic as stent material and manufacturer method also play a role. For example, one study found large differences between open cell stents in flexibility and radial force [9], while another found inferior scaffolding capacities for the closed cell Wallstent compared to the closed cell Xact and Cristallo Ideale (center part) due to the Wallstents’ braided wires which are capable of moving [3].

Second, none of the included papers drew comparisons between their outcomes and device characteristics as provided by the manufacturers. We have demonstrated that, except for foreshortening, these comparisons with manufacturer data are impossible to make due to different measurement techniques or some manufacturers’ reluctance to share their methodologies. Therefore, even after data querying from manufacturers, it is difficult to value the data provided. Translating these results to everyday clinical practice, it is currently impossible to achieve adequate patient-device matching as we cannot adequately compare carotid stents and are thus unable to test the manufacturers’ claims regarding their devices.

Third, multiple definitions exist for most of the stent characteristics, as for example for flexibility and conformability three different definitions are currently in use by the six stent manufacturers (Supplementary material, file 4). This illustrates the need for a universal definition of stent behavior. The final result of stent behavior is a complex interplay between characteristics of the stent itself and characteristics of the in vivo environment in which the stent is inserted, such as vessel wall characteristics and blood flow hemodynamics. This probably explains the difficulty in standardizing measurement methods and leads to unwanted but inevitable differences in definition.

### Recommendations

In order to enable comparisons between carotid stents, several recommendations are to be made. First, manufacturers are recommended to provide the device characteristics in the product description, accompanied by the applied testing methods. Second, in order to facilitate comparisons between devices and papers, it is advisable to choose one definition for each characteristic, to indicate a uniform testing methodology, and a standard for reporting outcomes. This facilitates clinicians in their choice for a specific device depending on the characteristics of the individual patient.

## Conclusion

A variety of methodologies and outcome measures is used to quantify carotid artery stent characteristics, which hampers comparisons between published studies, and between the literature and data as provided by device manufacturers. Future studies are encouraged to synchronize methodologies and outcome measures, and manufacturers are encouraged to increase transparency of applied testing methodologies and outcomes. This facilitates accurate comparisons between stents, adequate appreciation of manufacturers’ data, and improves patient-tailored device selection.

## Electronic supplementary material

Below is the link to the electronic supplementary material.

Supplementary material 1 (DOCX 34 kb)
