# Supplementary material for: (In)comparability of Carotid Artery Stent Characteristics: A Systematic Review on Assessment and Comparison with Manufacturer Data
Source: Cardiovasc Intervent Radiol. 2020 May 14;43(10):1430–7. doi: 10.1007/s00270-020-02499-1 (PMC7524852; doi:10.1007/s00270-020-02499-1)
Supplement: Supplementary file 1 — Supplementary material 1 (DOCX 34 kb) [file 270_2020_2499_MOESM1_ESM.docx]

**Supplementary material**

[Supplementary file 1. Search string 2](#_Toc37185703)

[Supplementary file 2. Websites of manufacturers 3](#_Toc37185704)

[Supplementary file 3. Contact form manufacturers 4](#_Toc37185705)

[Supplementary file 4. Manufacturer data 6](#_Toc37185706)

[Table 1. Vessel anatomy adaptability (conformability) and flexibility 6](#_Toc37185707)

[Table 2. Radial force and outward pressure 7](#_Toc37185708)

[Table 3. Visibility 8](#_Toc37185709)

[Table 4. Foreshortening 9](#_Toc37185710)

[Table 5. Scaffolding 10](#_Toc37185711)

[Table 6. Side-branch preservation 11](#_Toc37185712)

# Supplementary file 1. Search string

| Carotid artery | Stenting | Characteristics | In-vitro |
| --- | --- | --- | --- |
| Carotid[tiab] OR carotis[tiab])) | Stents[MeSH] OR stent*[tiab] OR device[tiab] | Characteristic*[tiab] OR propert*[tiab]  “Radial force”[tiab] OR “radial resistive force”[tiab] OR “radial recoil”[tiab] OR strength[tiab]  “Outward expansion”[tiab] OR “outward force”[tiab]  Scaffold*[tiab]  “Wall coverage”[tiab] OR “plaque coverage”[tiab]  “Anatomy coverage”[tiab] OR “vessel coverage”[tiab]  Conformability[tiab] OR adaptability[tiab] OR compliance[tiab]  Flexibility[tiab] OR torsion[tiab] OR bending[tiab]  Foreshortening[tiab]  (“Side-branch”[tiab] OR (externa[tiab] OR external[tiab])) AND (preservation[tiab] OR conservancy[tiab] OR conservation[tiab] OR safeguarding[tiab] OR safety[tiab] OR security[tiab])  Visibility[tiab] | “In vitro techniques”[MeSH]  “In-vitro”[tiab]  “Ex-vivo”[tiab]  Experiment*[tiab] |
|  |  |  |  |
| The columns were linked with “AND”, while subsequent rows were linked with “OR”. | | | |

# Supplementary file 2. Websites of manufacturers

Abbott – Acculink: <https://www.cardiovascular.abbott/us/en/hcp/products/peripheral-intervention/rx-acculink-carotid-stent-system.html>

Abbott – Xact: <https://www.cardiovascular.abbott/us/en/hcp/products/peripheral-intervention/xact-carotid-stent-system.html>

Cordis – Precise: <https://www.cordis.com/en_us/endovascular/intervene/self-expanding-stents/precise-pro-rx-carotid-stent.html>

Boston Scientific – Wallstent: <https://www.bostonscientific.com/en-US/products/stents--vascular/wallstent-endoprosthesis.html>

Medtronic – Protégé: <https://www.medtronic.com/us-en/healthcare-professionals/products/cardiovascular/peripheral-biliary-stents/protege-rx.html>

Terumo corp. – Roadsaver: <https://www.terumo-europe.com/en-emea/Products/Roadsaver%C2%AE-Carotid-Artery-Stent>

InspireMD – CGuard: <http://www.inspiremd.com/en/product/cguard/>

Gore – Gore carotid stent: <https://www.goremedical.com/products?f%5B%5D=product_type%3APeripheral&f%5B%5D=product_type%3AGrafts&q=>

Cardiatis – Cardiatis flow modulator: <http://www.cardiatis.com/?page_id=7>

NB. Associated brochures and instructions for use were also scrutinized.

# Supplementary file 3. Contact form manufacturers

Name:

Function:

Company:

1. Please fill in the requested information for the [*specific*] stent:

| Category | Specific characteristic* | Definition* | Used measurement method | Outcome | Unit |
| --- | --- | --- | --- | --- | --- |
| *Example: A* | *Radial force* | *The outward pressure that the stent can offer whilst retaining its diameter.* | *The parallel plate method whereby the stent was compressed by one third of its fully expanded diameter. The used force was measured using the Tinius Olsen 50N machine.* | *1.212±0.81* | *Newton (N)* |
| A | Radial force |  |  |  |  |
| B | Scaffolding |  |  |  |  |
| C | Vessel anatomy adaptability |  |  |  |  |
| D | Foreshortening |  |  |  |  |
| E | Side-branch preservation |  |  |  |  |
| F | Visibility |  |  |  |  |

*The literature uses different terms that differ slightly from each other, but do provide the same information about a stent. For example, radial force and outward expanding force both say something about how well the stent is resistant to outside forces. That is why we have categorized the stent properties (A-F). In the table below we have briefly summarized which synonyms we know, yet you may use other terms. However, we do like to hear the term(s) and definition(s) used by you.

| Category | Synonyms | Definition |
| --- | --- | --- |
| A | Radial force, outward expansive force/ strength/ recoil | The outward pressure that the stent can offer whilst retaining its diameter. |
| B | Scaffolding, wall/plaque coverage | The ability of the stent to hold an atherosclerotic plaque in place. |
| C | Vessel anatomy adaptability, conformability, flexibility, bending/ torsion stiffness | The ability of the stent to adjust its form to vessel tortuosity. |
| D | Foreshortening | The difference in stent length before and after deployment. |
| E | Side-branch preservation | The capacity of the stent to allow sufficient perfusion of side branches. |
| F | Visibility | The ability of the stent to provide sufficient opacity for stent positioning, deployment and post-procedure imaging. |

# Supplementary file 4. Manufacturer data

## Table 1. Vessel anatomy adaptability (conformability) and flexibility

| Stent | Definition provided by manufacturer | Synonyms used by manufacturer | Measurement method | Outcome | Unit |
| --- | --- | --- | --- | --- | --- |
| Wallstent | The ability of the stent to adjust its form to vessel tortuosity. | Bending stiffness | Conformability was assessed at the stent labeled diameter. Stents were tested for bending stiffness using a pure bend test apparatus, in a controlled 37°C environment. | 5.366* | Torque Nmm |
| Precise | Stent contourability and continuous wall apposition. | - | - | - | - |
| Protégé | see Wallstent | Flexibility | The flexibility of the stent is measured using a 3-point bend test on an Instron or MTS machine. | 16.3 (mean) | gf/mm |
| CGUARD | see Wallstent | - | Reference to conference presentation.(Wissgott 2016) | Outstanding adaptability | - |
| Roadsaver | The ability of the stent to adjust its form to vessel tortuosity without kinking. | - | Stents are bent to a diameter of 4mm and visually verified to be kink-free, to measure the kink resistance. Results were recorded as attribute data. | At 4mm, all units were kink-free | pass/fail |
| Gore | - |  | - | - | - |

Abbreviations: -, no data provided; gf, gram-force; mm, millimeters; N, Newton.

Footnotes: * data given for 10x31mm stent.

## Table 2. Radial force and outward pressure

|  | Stent | Definition provided by manufacturer | Synonyms used by manufacturer | Measurement method | Outcome | Unit |
| --- | --- | --- | --- | --- | --- | --- |
| radial force | Precise | The ability of a stent to withstand external forces. | Radial resistance force, hoop strength | Stents were deployed in center of a hoop strength fixture consisting of a compliant Dynatek tube and placed in a pressure device. Collapse pressure was measured (until complete collapse). | 1.72 | N |
| unknown | Wallstent | The outward pressure that the stent can offer whilst retaining its diameter. | - | Stents were tested with U-block, V-block, tapered U-block and V-block zone methods. Different methods were used to characterize the force properties in different test configurations. | Force V-block: 3.45* | Max force in gf/mm |
| outward pressure | Protégé | see Wallstent | Chronic outward force | Iris head method: stent was compressed uniformly around circumference down to catheter diameter and then expanded back to 1mm less than the labeled diameter. Force at that diameter was measured by the Machine Solutions tester. | 0.075 (mean) | N/mm^2^ |
|  | CGUARD | see Wallstent | - | Reference to conference presentation.(Wissgott 2016) | 2.28 | N/mm |
|  | Roadsaver | - | Outward expansive force, strength, recoil | Radial force is measured using Radial Force Tester RX550, which collects data from 12 product contact points and provides uniform radial measurement. | 235 ± 18^$^ | gf |
| - | Gore | - | - | - | - | - |

Abbreviations: -, no data provided; gf, gram-force; mm, millimeters; N, Newton.

Footnotes: * data given for 6x22mm stent, ^$^ data given for 6mm target vessel.

## Table 3. Visibility

| Stent | Definition provided by manufacturer | Measurement method | Outcome | Unit |
| --- | --- | --- | --- | --- |
| Wallstent | The ability of the stent to provide sufficient opacity for stent positioning, deployment and post-procedure imaging. | Fluoroscopy images were used to calculate the radiopacity of the stents. | 0.00540 | MPA |
| Precise | Visibility of the stent under fluoroscopy. | - | The delivery system consists mainly of an inner shaft and outer sheath with radiopaque markers. There are no markers on the stent. | - |
| Protégé | see Wallstent | The visibility is assessed by viewing the stent using fluoroscopy in animal studies. | 10 Tantalum spheres (5 on each end) are riveted within Nitinol hoops at the ends of the stent. | No. of Tantalum markers |
| CGUARD | see Wallstent | - | Clearly visible even on low radiation settings (data on file). | - |
| Roadsaver | see Wallstent | Radiopacity was graded in an animal study, on a rating scale of performance 1-3: 1, unacceptable; 2, acceptable; 3, excellent. | 2.65 ± 0.056 | - |
| Gore | - | - | - | - |

Abbreviations: -, no data provided; MPA, megapascal.

## Table 4. Foreshortening

| Stent | Definition provided by manufacturer | Measurement method | Outcome | Unit |
| --- | --- | --- | --- | --- |
| Wallstent | The difference in stent length before and after deployment. | The stent was deployed into a glass tube with inner diameter 1mm smaller than the labeled diameter. Shortening was calculated:  100 * ((length_constrained_ – length_parent_) / length_constrained_) | 49 | % |
| Precise | The change in the axial length of the stent during expansion. | Pictures taken at 10x magnification. | -≤8 | % |
| Protégé | The difference in stent length before (within the catheter) and after (in the vessel) deployment. | The stent length is measured in the catheter and then the stent is deployed into a mock artery. The length of the stent is then measured in the mock artery and then compared to the length of the stent in the catheter. | 0 | mm |
| CGUARD | see Wallstent | Calibrated measurement tool. | <6 | % |
| Roadsaver | see Protégé | The difference in loaded length of the stent (Lc) and final stent lengths (L) in target vessels is measured. Shortening was calculated:  100 * ((Lc – L) / Lc) | 27* | % |
| Gore | Percent length change. | Length on delivery system vs. length after deployment in glass model in 37°C water bath. | <9^$^ | % |

Abbreviations: %, percentage; mm, millimeters.

Footnotes: * data given for 7mm target vessel, ^$^ data given for straight stent.

## Table 5. Scaffolding

| Stent | Definition provided by manufacturer | Measurement method | Outcome | Unit |
| --- | --- | --- | --- | --- |
| Wallstent | The ability of the stent to hold an atherosclerotic plaque in place. | The stent was deployed in a glass tube with inner diameter 1mm smaller than the labeled diameter. The tube with stent was scanned using a Visicon inspection system, resulting in a ‘flat’ bitmap representation of the stent under backlight. Each image was analyzed for cell area, equivalent diameter, inscribed circle scaffolding index, and scaffolding uniformity. The metal-to-artery ratio was also calculated. | Cell area: 1.69*  Max cell diameter: 1.68*  Metal-artery ratio: 15* | Cell area: mm^2^  Max cell diameter: mm  Metal-artery ratio: % |
| Precise | Micromesh design, small cells, hybrid/ optimal cell design, short struts. | Pictures taken at 10x magnification. | 8.2 | mm^2^ |
| Protégé | see Wallstent | Percentage of metal-to-artery where the stent touches the vessel. A CAD model of the stent is used to determine the surface area of the outer diameter of the stent. That value is divided by the vessel area. | 29 | % |
| CGUARD | The material and construction giving the stent its ability to maintain the lumen of the artery and give good apposition to the intima. | GPD^5^ | Scaffold thickness: 240  MicroNet fibre: ≈ 20  Pore size: 150-180 | μm |
| Roadsaver | see Wallstent | To measure the metal surface area (MSA) % and pore cell size diameter of the stent. | MSA: 39±0.4^$^  Pore size: 536±30 | MSA: %  Pore size: μm |
| Gore | Diameter of fitted circle. | Nominal specification. | ePTFE lattice: 500 | μm |

Abbreviations: %, percentage; mm, millimeters; μm, micrometers; MSA, metal surface area.

Footnotes: * data given for 10x31mm stent, ^$^ data given for 7mm target vessel, ^5^ scaffold constructed of an open cell design nitinol cut tubular shape covered on the outside by a MicroNet® PET knitted jacket.

## Table 6. Side-branch preservation

| Stent | Definition provided by manufacturer | Measurement method | Outcome | Unit |
| --- | --- | --- | --- | --- |
| Wallstent | The capacity of the stent to allow sufficient perfusion of side branches. | Metal-to-artery ratio. See Table A5. | - | - |
| Precise | see Wallstent | GPD* | - | - |
| Protégé | The ability of the stent to allow flow throughout the body of the stent. | Measurement tool used for scaffolding (metal coverage). See Table A5. | - | - |
| CGUARD | see Wallstent | Reference to conference presentation.(Musialek et al. 2016) Number of external carotid arteries patent at 12 months. | 97 | % |
| Roadsaver | see Wallstent | To evaluate side-branch flow with chronic animal lab study. The patency of the blood flow was graded on a rating scale of performance 1-3: 1, unacceptable; 2, acceptable; 3, excellent. | all units with side-branches rated a 3 at 1-year follow-up | - |
| Gore | Patency of ECA in clinical use. | Reference to Gore SCAFFOLD clinical study. | <9^$^ | % |

Abbreviations: %, percentage; mm, millimeters; μm, micrometers; GPD; general product description; MSA, metal surface area.

Footnotes: * Autotapering design follows the vessel wall for enhanced conformability and wall apposition in the bifurcation, preserving complex angulations, and maintaining original wall anatomy.
